# A bladder cancer patient-derived xenograft displays aggressive growth dynamics in vivo and in organoid culture

**DOI:** 10.1038/s41598-021-83662-7

**Published:** 2021-02-25

**Authors:** Elise Y. Cai, Jose Garcia, Yuzhen Liu, Funda Vakar-Lopez, Sonali Arora, Holly M. Nguyen, Bryce Lakely, Lisha Brown, Alicia Wong, Bruce Montgomery, John K. Lee, Eva Corey, Jonathan L. Wright, Andrew C. Hsieh, Hung-Ming Lam

**Affiliations:** 1 Division of Human Biology, Fred Hutchinson Cancer Research Center, Seattle, WA USA; 2 Department of Urology, University of Washington School of Medicine, Seattle, WA USA; 3 Department of Pathology, University of Washington School of Medicine, Seattle, WA USA; 4 Department of Medicine, University of Washington School of Medicine, Seattle, WA USA; 5 Division of Public Health Sciences, Fred Hutchinson Cancer Research Center, Seattle, WA USA

**Keywords:** Cancer models, Cancer, Urological cancer, Bladder cancer

## Abstract

Bladder cancer is among the most prevalent cancers worldwide. Currently, few bladder cancer models have undergone thorough characterization to assess their fidelity to patient tumors, especially upon propagation in the laboratory. Here, we establish and molecularly characterize CoCaB 1, an aggressive cisplatin-resistant muscle-invasive bladder cancer patient-derived xenograft (PDX) and companion organoid system. CoCaB 1 was a subcutaneous PDX model reliably transplanted in vivo and demonstrated an acceleration in growth upon serial transplantation, which was reflected in organoid and 2D cell culture systems. Transcriptome analysis revealed progression towards an increasingly proliferative and stem-like expression profile. Gene expression differences between organoid and PDX models reflected expected differences in cellular composition, with organoids enriched in lipid biosynthesis and metabolism genes and deprived of extracellular components observed in PDXs. Both PDX and organoid models maintained the histological fidelity and mutational heterogeneity of their parental tumor. This study establishes the CoCaB 1 PDX and organoid system as companion representative tumor models for the development of novel bladder cancer therapies.

## Introduction

Urothelial carcinoma of the bladder is among the most common malignancies in the world^1^. Approximately 70% of bladder cancer patients have non-muscle invasive disease at diagnosis, but the recurrence rate is 60% at two years and risk of progression to invasive disease is high (40–70%)^2^. The remaining 30% of patients have the more aggressive muscle-invasive bladder cancer (MIBC) at diagnosis. For MIBC, the standard of care is neoadjuvant cisplatin-based chemotherapy followed by radical cystectomy. Despite optimal management, recurrences are common^3^ and second-line chemotherapy regimens have had limited success^4^.

A critical limitation in understanding bladder cancer progression and an impediment to targeted therapy development is the lack of well-characterized representative preclinical model systems. Genetic profiling has uncovered complex tumor heterogeneity that likely underlies variability in the therapeutic response^5–7^, necessitating laboratory models that can faithfully reproduce in vivo tumor phenotypes. Currently, few MIBC models have undergone thorough characterization to assess their fidelity to patient tumors, especially upon propagation in the laboratory. Cell lines established from human tumors are widely employed in preclinical studies due to their ease of use and reliable growth, but they are generally insufficient clinical models. Marked differences between the in vitro culture and the in vivo tumor microenvironment raises concerns that these lines do not fully represent tumors in the patients^8,9^. The 2D monolayer culturing process causes significant genetic drift away from the original tumor and results in a high degree of homogeneity, which does not reflect the true heterogeneity of primary tumors and negatively affects our ability to probe therapeutic resistance. Patient-derived xenograft (PDX) models of bladder cancer have improved our ability to model primary tumor architecture, microenvironment interactions, and genetic heterogeneity^10,11^. However, PDX models can be challenging and resource-intensive to establish and maintain. Recently, patient-derived bladder cancer organoids have been developed as a highly adaptable and scalable model system^12,13^. Organoids are superior to 2D monolayer culture because they can mirror the in vivo response to chemotherapy drugs in solid tumors such as bladder cancer^14,15^. PDX and organoid systems are promising tools for preclinical testing of novel therapeutics and for precision medicine. However, to validate their utility as clinical models, they require extensive functional and molecular characterization to establish their fidelity to human bladder cancers.

Human tumors exhibit progressive phenotypic changes as a reflection of their mutational heterogeneity^6,16^. Subclones evolve dynamically in space and time, leading to emergent features such as drug resistance and metastasis. Existing PDX and organoid models have also demonstrated clonal evolution throughout passaging and culture, though such changes do not always reflect the genetic composition of the parental tumor^17^. To date, there has been limited comparison between bladder cancer PDX and organoid models to dissect the evolution of their growth dynamics and molecular profiles relative to their parental tumor. Here, we established and characterized the phenotypic and transcriptomic progression of a cisplatin-resistant MIBC PDX (CoCaB 1) and an organoid system derived from the PDX. We revealed that accelerated growth upon passaging was critical for shaping the biological landscape of PDXs and their derivative organoids. In vivo propagation selected for more proliferative and stem-like phenotypes, consistent with observations in breast and hematopoietic cancers^18,19^. This aggressive growth advantage was maintained in ex vivo organoid models. Both the PDX and organoid models successfully maintained the histological fidelity and mutational heterogeneity of their parental tumor. Our study highlights the value of PDXs and organoids as representative tumor models for the development of novel bladder cancer therapies.

## Results

### Establishment of the CoCaB 1 PDX and organoid models

**Figure 1:**
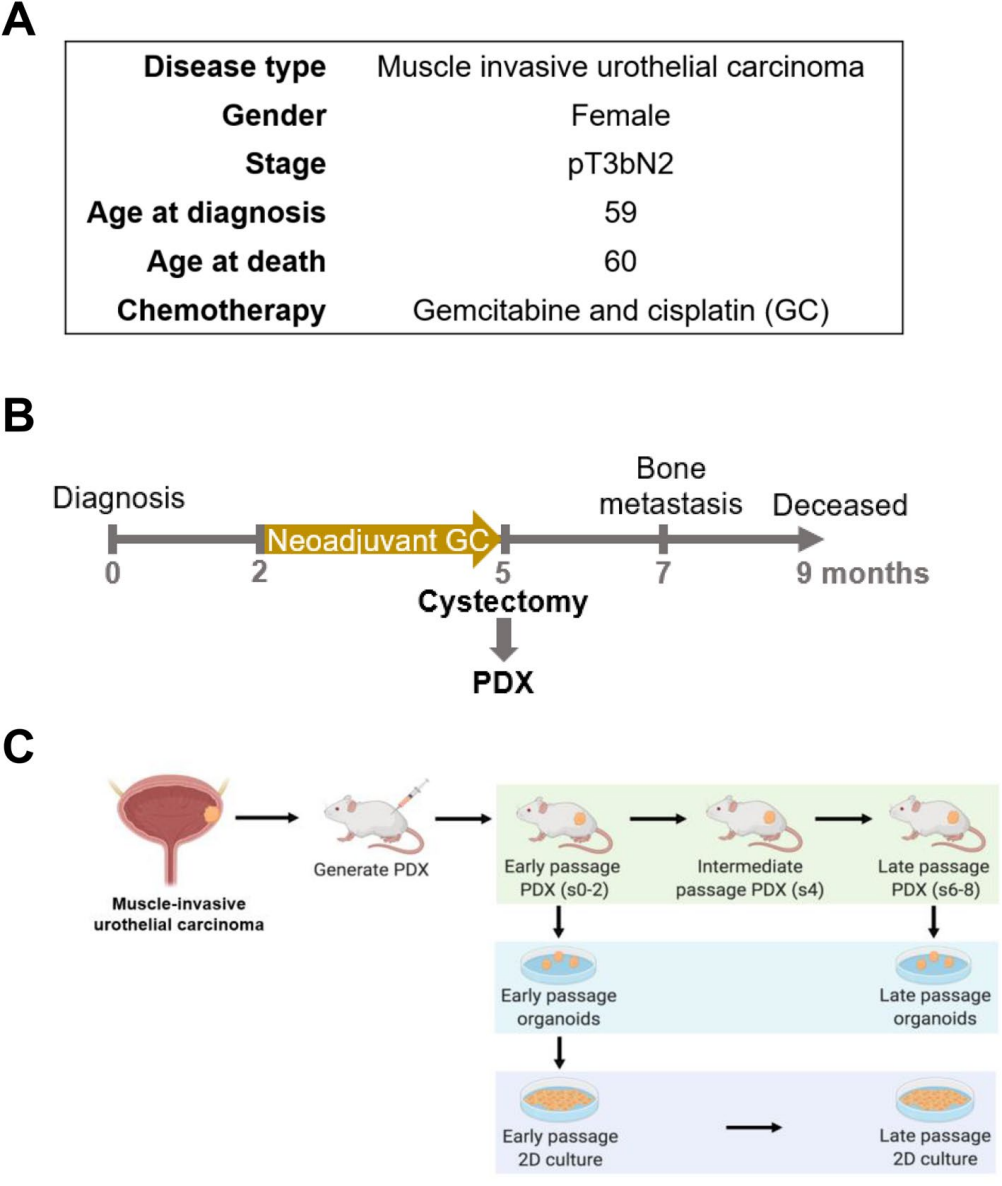
CoCaB 1 PDX patient characteristics. (**A**) Clinical information of the patient. (**B**) Timeline and clinical course of the disease. (**C**) Experimental design to establish laboratory models, created with Biorender.com.

A tumor from a MIBC patient refractory to cisplatin-based chemotherapy was used for ex vivo model establishment and growth dynamic studies (Fig. 1A). The patient has a very aggressive disease course, with 9 months from diagnosis to death Fig. 1B). After standard neoadjuvant treatment with gemcitabine and cisplatin, cystectomy was performed where residual pT3bN2 tumor was present. Excised urothelial carcinoma tissue was implanted subcutaneously into two SCID mice to generate CoCaB 1 PDXs (Fig. 1B), and both mice developed tumors. CoCaB 1 was serially passaged in mice, and successfully cryopreserved and resurrected, allowing long-term storage for future studies. Upon serially transplanting tumors to 10 passages, we observed the efficiency of developing CoCaB 1 PDX was 100% (n = 34/34). Next, we performed CoCaB 1 functional and molecular characterization at different passages (early: s0-2, intermediate: s4, late: s6-8; ‘s’ refers to SCID mouse) to inform model selection for biological and preclinical studies. Representative early and late passage PDXs were collected for organoid establishment, followed by 2D culture to establish models allowing genetic manipulation and high-throughput screening (Fig. 1C).

### PDX recapitulates primary tumor features and displays aggressive growth dynamics

**Figure 2:**
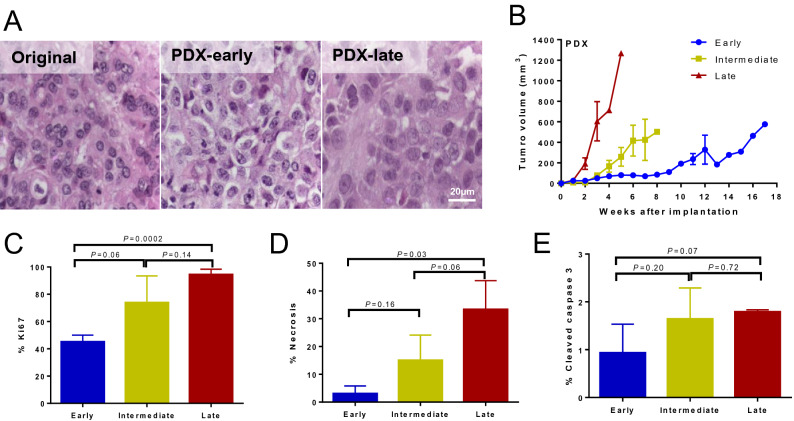
Bladder cancer PDX CoCaB 1 grows faster in late vs. early passages. (**A**) Representative IHC staining showing conserved high-grade urothelial carcinoma histology among original tumor, early, and late passages of PDX. Magnification 200×. Scale bar represents 20 µm. (**B**) CoCaB 1 PDX grows progressively faster in later passages (s6-8 late, s4 intermediate, s0-2 early; n = 8–10 for early and late passages, n = 4 for the intermediate passage). (**C**–**E**) Late passage PDX showed (**C**) an increase in the Ki67 proliferation index and (**D**) an increase in necrosis, but (**E**) no change in cleaved caspase 3 vs. early passage PDX.

To functionally examine the fidelity of the CoCaB 1 PDX as a MIBC model, we first compared the histology of each PDX passage to the parental tumor (Fig. 2A). Of the 16 PDXs collected for histological evaluation (out of 34 animals), all (16/16) successfully conserved the histology of the original high-grade and poorly differentiated urothelial carcinoma, from early through late passages (Fig. 2A). In vivo, the latency of PDX establishment progressively decreased upon passaging, from time to initial growth of 9 weeks in early passages to 2 weeks in late passages (Fig. 2B). To investigate how passaging affects growth dynamics, we tracked tumor growth in early, intermediate, and late passage PDXs. Tumor growth rate progressively accelerated during passaging, with late passage PDXs rapidly reaching endpoint tumor size (1000 mm^3^) only 5 weeks post-implantation (Fig. 2B). To dissect the cellular behaviors underlying increased tumor growth in late passage PDXs, we examined proliferation and cell death using histology and immunohistochemistry (IHC). Ki67 proliferation index was positively correlated with passage number (Spearman R = 0.804, *P* = 0.001), increasing from 45% in early passage to 95% in late passage PDXs (*P* = 0.0002, Fig. 2C and Supplementary Fig. S1A). Accelerated proliferation was accompanied by an increase in necrosis in late passage PDXs (*P* = 0.03, Fig. 2D), but no change in apoptosis was observed as measured by cleaved caspase 3 (Fig. 2E). Therefore, the accelerated tumor growth found in late passage PDXs is supported by a significant elevation in proliferation rate rather than a decrease in cell death, while maintaining the histological fidelity of high-grade urothelial carcinoma as the parental tumor.

### Organoid models reproduce PDX growth dynamics and evolution

**Figure 3:**
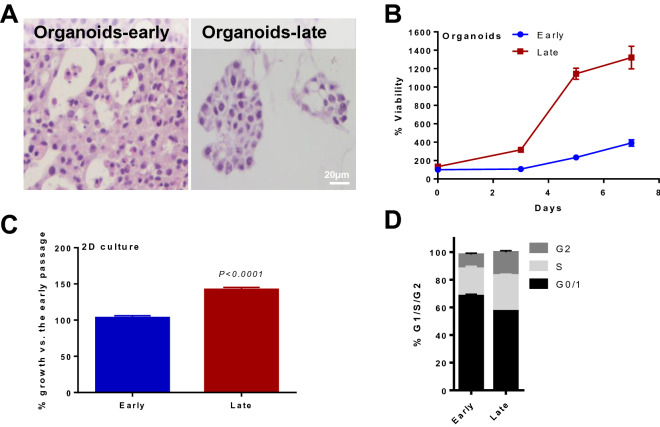
CoCaB 1 PDX-derived organoids reproduce CoCaB 1 PDX progressive growth phenotypes. (**A**) Representative IHC staining showing conserved high-grade urothelial carcinoma histology in early and late passage organoids. Magnification 200x. Scale bar represents 20 µm. (**B**,**C**) Late passage (**B**) organoids (s2 early, s9 late; n = 4–8) and (**C**) cultured cells (s2 early, s10 late; n = 6) grow faster than early passage organoids and cells. (**D**) In organoids, late passages display an increase proportion of cells in the S phase of the cell cycle.

We generated CoCaB 1 organoids from representative early and late passage PDXs in order to further probe their growth dynamics and cellular fidelity as companion tumor models. Both early and late PDX passages were successfully developed into organoid cultures, which could be cryopreserved and resurrected. We have attempted directly adapting patient tumors to establish organoid culture, however organoids were not formed. PDX-derived organoids maintained the high-grade urothelial carcinoma histology of their parental PDXs and of the original patient tumor (Figs. 2A and 3A, and Supplementary Fig. S1B), and displayed cisplatin insensitivity (IC50 = 6.3 µM, Supplementary Fig. S1C) similar to cisplatin-resistant bladder cancer cell lines^30^. Organoids also faithfully recapitulated PDX growth phenotypes, with late passage organoids undergoing accelerated proliferation, measured by the MTS assay, compared to early passage organoids (Fig. 3B). To corroborate this finding, we generated 2D cultures derived from representative early passage organoids (s2) and passaged in vitro to a late passage (s10, Supplementary Fig. S1B). In late passage 2D culture, we found a 50% increase in growth when compared to the early passage (*P* < 0.0001, Fig. 3C), consistent with the growth acceleration identified in PDX and organoids (Fig. 2B,C and Fig. 3B). Furthermore, the enhanced proliferation in late passage organoids is supported by an increased % of cells in S phase of the cell cycle and decreased % of cells in the G0/G1 phase (Fig. 3D). Overall, these studies demonstrate that CoCaB 1 organoids and 2D cultures preserve the growth phenotype of the originating PDX, providing faithful in vitro models for future studies.

### Transcriptome analysis reveals evolution towards an aggressive growth state

**Figure 4:**
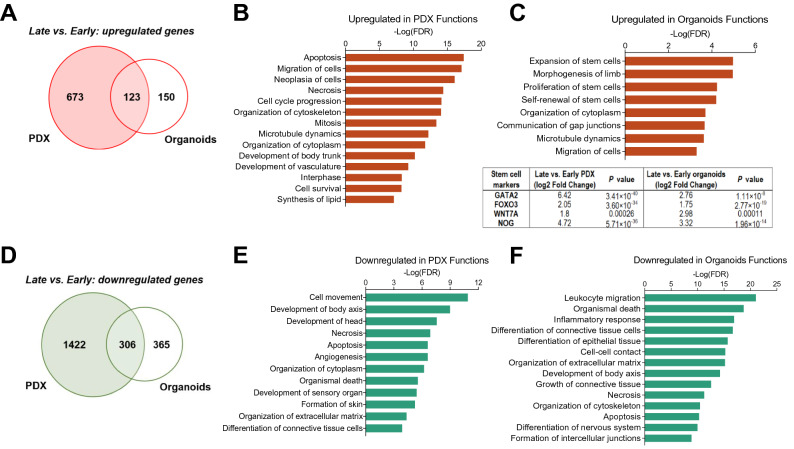
Late passage PDX and organoids upregulate cell cycle and stem cell regulators. (**A**) Upregulated genes in late passage vs. early passage PDX and organoids. (**B**,**C**) Ingenuity pathway analysis of genes upregulated in late passage (**B**) PDX and (**C**) Top panel: organoids reveals enrichment of cell cycle, stem cell, and cell migration regulators. Bottom panel: Stem cell marker expression in late vs. early PDX and organoids by RNAseq. (**D**) Downregulated genes in late passage vs. early passage PDX and organoids. (**E**,**F**) Ingenuity pathway analysis of genes downregulated in late passage (**E**) PDX and (**F**) organoids reveals enrichment of cell death, cell adhesion, and differentiation regulators.

We next performed transcriptome profiling to understand the molecular mechanisms associated with aggressive growth evolution during passaging in both PDX and organoid models. Gene expression in early and late stage PDXs and organoids were compared by edgeR (|Log_2_FC|≥ 1, FDR < 0.01)^22^ after removal of mouse sequences. In comparing late vs. early passage PDXs, we found transcriptional upregulation of 796 genes (Fig. 4A, Table S1). Ingenuity pathway analysis (IPA) of molecular functions revealed enrichment of proliferation, mitosis, and cell migration genes, indicating dysregulation of growth pathways (Fig. 4B). Similarly, when comparing late vs early passage organoids, we found 273 upregulated genes (Fig. 4A) that were enriched for stem cell proliferation and self-renewal, suggesting a more stem-like expression profile in late passages (Fig. 4C top panel, Table S2). Notably, known stem cell renewal regulators *Gata2, Foxo3, Wnt7a,* and *Nog*^31–34^ were all significantly upregulated in both late passage PDXs and organoids (Fig. 4C bottom panel, Table S1 and S2). There were 123 upregulated genes shared between PDX and organoid passage analysis (Fig. 4A); however no pathway was significantly enriched based on the limited number of overlapping genes. Concordantly, we found that both late passage PDX and organoid systems exhibited downregulation of genes involved in cell differentiation, tissue development, and cell death (Fig. 4D–F, Tables S1 and S2). These pathways were consistently enriched in the 306 downregulated genes shared between PDX and organoid analyses (Fig. 4D and Supplementary Fig. S1D), further demonstrating that passaging selects for poorly differentiated and fast-growing phenotypes. To gain insight into potential subtype plasticity^12^ in CoCaB 1 PDX and organoid models, we performed consensus subtype analysis^26^ based on the RNAseq data. The patient tumor was successfully classified as Basal/Squamous (Ba/Sq) subtype, which were largely consistent between PDX and organoids, and conserved from early to late passages (Supplementary Fig. S2A). Despite late passage organoids were assigned to neuroendocrine-like (NE-like) subtype, the subtype separation level and the expression of NE markers were low (Supplementary Fig. S2B), suggesting it is likely too close to other consensus classes to be confidently assigned as NE-like. These findings indicate that CoCaB 1 organoids recapitulate PDX growth behavior by maintaining key components of the PDX expression profile. In vivo selection for more aggressive phenotypes during passaging may mirror clinical tumor evolution, serving as a powerful tool for therapy development against advanced chemotherapy-resistant disease.

### Transcriptome differences between PDX and organoid models reflect differences in cellular composition

**Figure 5:**
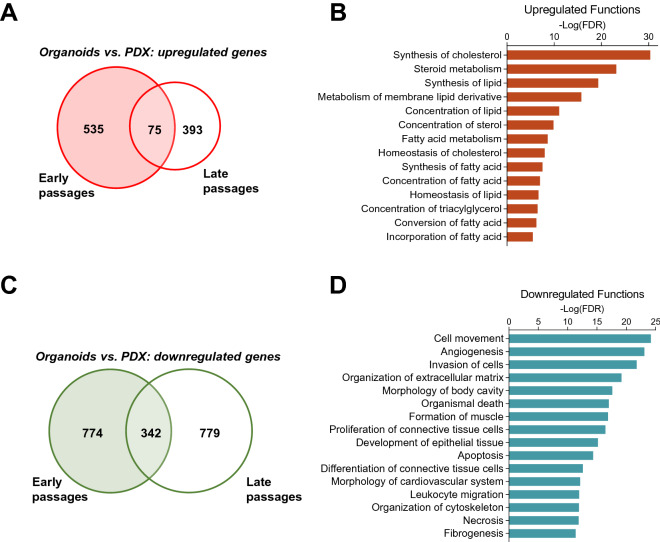
Transcriptional changes between PDX and organoids models reflect differences in cell composition. (**A**) Upregulated genes in organoids vs. PDX in early and late passages. (**B**) Ingenuity pathway analysis of genes upregulated in organoids reveals enrichment of lipid synthesis and metabolism genes. (**C**) Downregulated genes in organoids vs. PDX in early and late passages. (**D**) Ingenuity pathway analysis of genes downregulated in organoids reveals enrichment of extracellular matrix genes.

To further evaluate organoids as an ex vivo model of in vivo (PDX) cellular behavior, we analyzed transcriptional differences between PDXs and their derivative organoids. First, we determined the extent of mouse stromal involvement in PDX or stromal retention in PDX-derived organoids by aligning RNAseq reads to both human and mouse genomes. PDXs contained approximately 90% human transcripts, with mouse stromal content accounting for the remaining 10% (Table 1), which remained constant through PDX late passages. In contrast, organoids contained > 99% human transcripts despite being derived from PDXs established in mice, reflecting the lack of mouse stroma in organoids. Next, we compared transcriptional differences between organoid and PDX models, specific to human sequences, by edgeR (|Log_2_FC|≥ 1, FDR < 0.01). In both early and late passages, organoids exhibited upregulation of lipid biosynthesis and metabolism genes when compared to PDXs (Fig. 5A,B, Tables S3 and S4). Increased lipid remodeling and cholesterol biosynthesis has been implicated in enhanced stem cell maintenance, proliferation, and tumorigenesis both in vivo and in organoids^35,36^. In our studies, lipid biosynthesis upregulation likely reflects the increased stem cell composition of organoids relative to stromal rich PDXs. Expectedly, in both early and late passages, organoids exhibited significant downregulation of angiogenesis, cell migration, extracellular matrix, and differentiation genes (Fig. 5C,D), consistent with the absence of the stromal compartment in organoids. Therefore, the transcriptional differences between PDX and organoids reflect inherent differences in cellular composition between the two model systems. While both PDXs and organoids display similar aggressive tumor growth phenotypes upon passaging, their unique cellular compositions offer each model a distinct role in the development of novel therapeutics.

**Table 1:** PDXs and organoids human sequence match by RNAseq.

	% hg38 (mean±SD)	P value
Patient	99.5	
PDX-EarlyƗ	89.4±4.3	
PDX-LateƗƗ	90.1±1.2	0.81Ɨ
Organoids	99.2±0.1	0.003Ɨ
		<0.001ƗƗ

### Serial transplantation preserves the mutational profile of the parental tumor

To determine the genetic fidelity of PDXs and organoids to patient tumors, we examined whether the mutational profile of the original tumor was preserved during model establishment and passaging. We manually curated RNAseq reads aligned to coding sequences of all genes with mutation frequency > 5% in TCGA MIBCs^5^ to identify missense mutations that are present in our original patient tumor and maintained throughout our PDX and organoid models. Across the 86 genes examined, we identified 33 maintained missense mutations whose mutational locus was catalogued in COSMIC, including TP53, KMT2D, EP300, ATM, FAT1, and ERBB2 (Table 2)^28^. The preserved mutations among the patient tumor, early through late passages of PDX, and organoids included mutations that are predicted to be pathogenic in cancers^29^, representing both homozygous (FAT1) and heterozygous mutations (ERBB2, Supplementary Fig. S3A,B). Notably, the D1399 residue of EP300 is critical for its catalytic activity as a cell cycle regulator and is frequently mutated in invasive bladder cancer subtypes^37–39^. This mutation is predicted to have a pathogenic score of 0.99 (range 0–1) and is conserved in the CoCaB 1 patient tumor, PDX and organoid models (Table 2). Thus, PDXs and organoids successfully preserve key pathological mutations of the patient tumor throughout passaging, serving as clinically relevant models for exploring targeted therapies.

**Table 2:** Missense mutations present in original patient tumor, preserved in CoCaB 1 PDX and organoid, and catalogued in COSMIC.

Gene	% mutated in TCGA MIBC	AA mutation	Het/Homo	COSMIC mutation ID	FATHMM prediction
*TP53*	48.10%	P72R	Homo	COSV52666208	Neutral (score 0.36)
		R110P	Het	COSV52668419	Neutral (score 0.12)
*KMT2D*	28.40%	R83Q	Het	COSV56410163	Pathogenic (score 0.79)
		P813L	Het	COSV56410262	Neutral (score 0.08)
*EP300*	15.30%	D1399G	Homo	COSV54326888	Pathogenic (score 0.99)
*ATM*	13.80%	D1853N	Het	COSV53728020	Pathogenic (score 0.98)
*FAT1*	12.40%	V482L	Homo	COSV71672022	Pathogenic (score 0.95)
		R1064G	Homo	COSV71675744	Pathogenic (score 0.97)
		Q2933P	Het	COSV71675402	Pathogenic (score 0.85)
		V3334A	Het	COSV71673573	Pathogenic (score 0.93)
		S3554A	Het	COSV71673371	Neutral (score 0.04)
		D4218G	Het	COSV104437749	Pathogenic (score 0.93)
*ERBB2*	12.10%	G776A	Het	COSV54062853	Pathogenic (score 0.98)
*PDE4DIP*	10.90%	R25L	Het	COSV57671760	Pathogenic (score 0.92)
		R1504Q	Het	COSV57679587	Pathogenic (score 0.76)
		R2291Q	Het	COSV57672336	Neutral (score 0.01)
*BRCA2*	9.50%	V2466A	Homo	COSV66451785	Neutral (score 0.00)
*MKI67*	8.70%	G1042S	Het	COSV64072617	Neutral (score 0.12)
		E1403V	Het	COSV64073379	Neutral (score 0.16)
		I2101T	Het	COSV64074379	Neutral (score 0.08)
		D2751N	Het	COSV104424640	Neutral (score 0.02)
*RNF213*	8.50%	C3008R	Het	COSV60411421	Neutral (score 0.06)
*ATR*	7.50%	Q2625L	Homo	COSV99377126	Neutral (score 0.38)
*SETD2*	6.80%	P1962L	Het	COSV57428846	n/a
*SPEN*	6.60%	L1091P	Het	COSV65359297	Neutral (score 0.02)
		N1856S	Het	COSV65369305	Neutral (score 0.13)
*ANKRD11*	6.30%	A971V	Het	COSV99970995	Neutral (score 0.12)
*APC*	6.10%	V1822D	Het	COSV57321643	Neutral (score 0.05)
*ATRX*	6.10%	E929Q	Homo	COSV64880404	Neutral (score 0.17)
*KMT2B*	5.60%	D2364G	Het	COSV55857349	Neutral (score 0.04)
*ZFHX3*	5.30%	H3611Y	Het	COSV51720325	Pathogenic (score 0.90)
*KNL1*	5.10%	A486S	Homo	COSV61150768	Neutral (score 0.15)
*PTPN13*	5.10%	Y2086D	Het	COSV57415600	Neutral (score 0.00)

## Discussion

We report the establishment and characterization of the CoCaB 1 PDX and organoid models of aggressive MIBC. These platforms successfully maintained the original tumor histology and demonstrated concordant growth phenotypes. Both PDX and organoids exhibited increased proliferation upon serial passaging. Transcriptome analysis revealed progression towards a more proliferative, stem-like, and invasive expression profile during passaging. Despite these transcriptional changes, our studies demonstrate that PDX and organoids faithfully maintain key pathological mutations identified in the primary tumor. Other genetic alterations that can arise during passaging, such as copy number alterations and indels^17^, may be underlying our observed transcriptional changes and require further genomic analysis. To our knowledge, this is the first study that functionally and molecularly characterizes PDX and organoid growth dynamics during passaging.

Efforts to advance precision medicine and develop targeted therapies for bladder cancer have been hampered by the lack of well-characterized and easily expandable laboratory models^40^. Despite differences in cellular composition and tissue architecture, we showed that PDXs and organoids have highly similar growth responses and represent two independent model systems with significant potential for accelerating the study of tumor biology and therapy. PDXs and organoids select for aggressive subclones during passaging, potentially modelling the evolution of aggressive disease in patients. Our finding that PDXs and organoids are biologically similar at equivalent passages provides a potential workflow for therapy development. Since high-throughput drug screening requires significant tissue quantities, but PDX models are resource-intensive to maintain, we can first employ organoids as an easily expandable model to conduct preliminary drug screening. Candidates can then be validated in PDX to examine tumor response involving its microenvironment^41^ and systemic responses.

High-throughput screening with subsequent *in vivo* testing in xenografts has been an important strategy in identifying response predictors and resistance mechanisms^42,43^. However, when shifting drug studies from organoids to PDX, several fundamental differences between organoids and PDX need to be noted. First, these models have differential drug penetrance that can significantly affect observed drug responses. For example, in organoids, doxorubicin has poor penetrance beyond the first 3 or 4 layers of cells within spheroids^44^, and spheroid size itself affects drug penetrance and cytotoxic response^45^. In contrast, drug administration in PDX models is usually systemic. Secondly, substantial differences in tumor microenvironment can affect drug response. While organoids can be generated by co-culturing tumor cells with cells comprising the tumor microenvironment (e.g. fibroblast, endothelial cells, etc.), their temporal and spatial distribution is likely to differ from those in PDXs and human tumors^46^.

PDXs and organoid biobanks can capture the broad genetic diversity of patient tumors, making them ideal for disease modeling and therapeutic screening^10–13^. However, the genetic evolution of laboratory models may differ from patient tumors in vivo, potentially complicating their fidelity. The inherent genomic instability of tumor cells can distance the genomic profile of models from the patient tumor^42^. As evidenced in our study, laboratory models demonstrate gene expression evolution during passaging that suggests selection of aggressive subclones or acquisition of de novo genetic lesions. Furthermore, PDXs can rapidly acquire copy number alterations that differ from those acquired during tumor evolution in patients^17^, likely due to difference in selection pressure. These genetic differences manifest as variations in clonal composition, potentially causing discrepancies in therapy response^47^. The extent to which clonal selection in MIBC laboratory models mirrors patient tumor progression remains unclear and requires generation and extensive characterization of bladder cancer biobanks. Nevertheless, the techniques employed here can be used to generate a large repertoire of patient-derived bladder cancer models amenable to cryopreservation, and genetic and pharmacologic studies. Notably, this strategy of establishing organoids from PDXs is very reliable (100% take rate based on six different CoCaB models; data not shown), suggesting that PDX-derived organoids are a more robust method of companion model establishment than deriving organoids directly from MIBC patient tumors^12^. PDX and PDX-derived organoids provide valuable new tools in the study of aggressive bladder cancer.

## Methods

### Patient specimens and clinical data

Tissue acquisition for research was performed in accordance with relevant guidelines and regulations, and approved by the University of Washington Human Subjects Division Institutional Review Board (IRB #39053). Tumors were acquired from a patient who underwent cystectomy and signed an informed consent. Patient information and associated clinical information was de-identified.

### Patient-derived xenograft (PDX) development

Animal procedures were performed in accordance with NIH and ARRIVE guidelines for in vivo studies carried out on animals, and approved by the University of Washington Institutional Animal Care and Use Committee. Male CB-17 SCID mice (aged 6–8 weeks; Charles River Laboratories, San Diego, CA) were implanted subcutaneously with 2 mm^3^ bladder cancer tumor pieces immersed briefly in Matrigel (# 356,237; Corning, Corning, NY). For transplantation, 2 mm^3^ PDX tumor pieces without Matrigel were used. Subcutaneous tumor growth was measured twice weekly using a digital caliper and volume was calculated using the formula: length x width x height × 0.5236. Animals were sacrificed when tumors exceeded 1000 mm^3^ or if animals became compromised. Established PDX was designated as CoCaB 1.

### 3D organoid development

CoCaB 1 PDX chunks were minced to 1 mm pieces with a sterile scalpel in PBS and incubated in 5 mg/ml Type II Collagenase (Gibco, Waltham, MA) in Advanced Dulbecco’s modified Eagle’s medium (DMEM)/F12 for 1 h at 37 °C, followed by 5 min further digestion with 0.25% trypsin. The tissue-cell mixture was passed through a 40um nylon mesh to obtain single cells. Twenty thousand cells were embedded in 50 µl Matrigel (Corning) and cultured in complete medium containing B-27 supplement, 1.25 mM N-Acetyl-L- cysteine (Sigma-Aldrich, St. Louis, MO), 50 ng/ml EGF (Peprotech, Rocky Hill, NJ), 200 nM A83-01 (Tocris Bioscience, UK), 500 ng/ml R-spondin1 (conditioned medium and R-spondin1 expressing plasmid provided by Yu Chen, Memorial Sloan Kettering Cancer Center), 10 µM Y- 27,632 (Sigma-Aldrich), and 100 ng/ml Noggin (Peprotech) in 24-well ultra-low attachment plates (Corning) as previously described^20^.

### Adaptation of organoids to 2D culture

Organoids were dissociated to single cells by enzymatic digestion of Matrigel using dispase (STEMCELL technologies) at 37 °C for 15 min followed by TrypLE (Gibco) at 37 °C for 5 min. Cells were subsequently seeded in tissue culture plates with DMEM-10 (DMEM supplemented with 10% fetal bovine serum, GlutaMAX (Gibco), and penicillin/streptomycin). Cells were grown and passaged at 70% confluency for at least three passages to establish cell lines.

### Cell viability assay

For the cisplatin dose–response curve, early passage CoCaB 1 organoids were treated with cisplatin for 48 h and cell viability was measured using CellTiter-Glo (Promega, Madison, WI). For the 2D culture, early and late passage CoCaB 1 cells were plated in DMEM-10 medium (5000 cells/well) in a 96-well plate. Cell viability was measured 96 h after seeding using CellTiter-Glo.

### Cell cycle analysis

CoCaB 1 organoids were starved in medium without EGF for 24 h and changed into complete medium with EGF for 72 h. Organoids were dissociated into single cells by 20 min incubation in TrypLE at 37 °C. Cells were washed in PBS and fixed in cold 70% ethanol for 30 min. Fixed cells were stained for 20 min in 50 ug/ml propidium iodide + 0.1% Triton X-100 + 200 ug/ml RNase in PBS. At least 10,000 cells per sample were acquired using BD LSR II Flow Cytometer System (BD Biosciences, San Jose, CA) and data was analyzed by FlowJo (BD Biosciences, San Jose, CA). Experiments were performed in triplicate and repeated.

### Histology and immunohistochemistry analysis

Formalin-fixed, paraffin-embedded patient tumor, PDXs, and organoids were sectioned at 5-µm and stained with hematoxylin and eosin for histologic evaluation by a genitourinary pathologist (F.V.L.). Immunohistochemistry analyses for Ki67 (Clone MIB-1, 1:100; Agilent Dako, Santa Clara, CA) and cleaved caspase 3 (Asp175, 1:100; Cell Signaling, Danvers, MA) were carried out as previously described^21^. Ki67 was assessed by the percentage of cells stained in each section. Cleaved caspase 3 was assessed using Image J by averaging the number of stained cells in 5 representative regions using the formula: positive cells/(positive + negative cells) × 100%. Hematoxylin and eosin staining was performed to histologically assess necrotic area. All evaluations were performed in a blinded fashion.

### RNA sequencing and bioinformatic analysis

RNA was extracted using RNA STAT 60 (Tel-Test, Friendswood, TX) and RNeasy Mini kit, followed by DNase digestion in solution prior to purification (Qiagen, Germantown, MD). RNA integrity number was determined using Agilent Bioanalyzer (Agilent, Santa Clara, CA). RNA-seq libraries were constructed from total RNA using TruSeq mRNA LT Sample Prep Kit according to the manufacturer’s protocol (Illumina, San Diego, CA). Barcoded libraries were pooled and sequenced using Illumina HiSeq 2500 system to obtain 50-bp paired-end reads (Illumina, San Diego, CA). Reads were aligned to human hg38 and mouse mm10 genomes using TopHat2^22^. Alignments matching hg38 with a higher fidelity than mm10 were retained, where fidelity is measured by the number of mismatches against the reference genome^23^. Gene counts were generated for each gene with HTSeq v0.6.1p1 using the "intersection-strict" overlapping mode^24^. Differential expression was conducted using the edgeR Bioconductor package in R, filtering for 3 counts/million in at least one sample and applying |log Fold Change|> = 1 and a significance level of 0.01 with Benjamin-Hochberg false discovery rate (FDR) adjustment^25^. Ingenuity pathway analysis (QIAGEN, https://www.qiagenbioinformatics.com/products/ingenuity-pathway-analysis) was performed on differentially expressed genes to identify enriched molecular and cellular functions. For each of the CoCaB 1 models, R package “consensusMIBC” was used to identify consensus subtypes^26^. The separation level (0–1) measures how a sample is representative of its consensus class, with 0 meaning too close to other consensus classes to be confidently assigned its consensus class label ,and 1 meaning a sample is very representative of its consensus class and very different from the other consensus classes. RNAseq data are deposited in the Gene Expression Omnibus database under the accession number GSE155007.

### Missense mutation analysis

Aligned sequences from RNAseq were loaded into the Integrative Genome Viewer^27^. Coding regions of genes with mutation frequency > 5% in TCGA MIBC5 were manually examined for homozygous or heterozygous missense mutations that were present in the original patient tumor and carried through early and late passage CoCaB 1 PDXs and organoids. Preserved missense mutations were probed for in COSMIC^28^ and classified as a somatic mutation if the mutational locus was catalogued in COSMIC. FATHMM-XF was used to predict pathogenic mutations^29^.

### Statistical analyses

Statistical comparisons between early vs. late passages in different models were conducted by Mann–Whitney test. Data are means ± SEM, except for in vitro cultures that were represented as means ± SD. For IPA analyses, a molecular function that displayed FDR < 5% was considered significantly enriched.

## Supplementary Information

Supplementary Information 1.

Supplementary Information 2.

Supplementary Information 3.

Supplementary Information 4.

Supplementary Information 5.

## Data Availability

The RNAseq datasets generated and analyzed during the current study are available in the Gene Expression Omnibus database under the accession number GSE155007.

## References

[1] Bray F (2018). Global cancer statistics 2018: GLOBOCAN estimates of incidence and mortality worldwide for 36 cancers in 185 countries. CA Cancer J. Clin..

[2] Chamie K (2013). Recurrence of high-risk bladder cancer: a population-based analysis. Cancer.

[3] Chang SS (2017). Treatment of non-metastatic muscle-invasive bladder cancer: AUA/ASCO/ASTRO/SUO guideline. J. Urol..

[4] Meeks JJ (2012). A systematic review of neoadjuvant and adjuvant chemotherapy for muscle-invasive bladder cancer. Eur. Urol..

[5] Robertson AG (2017). Comprehensive molecular characterization of muscle-invasive bladder cancer. Cell.

[6] Faltas BM (2016). Clonal evolution of chemotherapy-resistant urothelial carcinoma. Nat. Genet..

[7] Winters BR (2019). Genomic distinctions between metastatic lower and upper tract urothelial carcinoma revealed through rapid autopsy. JCI Insight.

[8] Nickerson ML (2017). Molecular analysis of urothelial cancer cell lines for modeling tumor biology and drug response. Oncogene.

[9] Goodspeed A, Heiser LM, Gray JW, Costello JC (2016). Tumor-derived cell lines as molecular models of cancer pharmacogenomics. Mol. Cancer Res..

[10] Pan C-X (2015). Development and characterization of bladder cancer patient-derived xenografts for molecularly guided targeted therapy. PLoS ONE.

[11] Jäger W (2015). Patient-derived bladder cancer xenografts in the preclinical development of novel targeted therapies. Oncotarget.

[12] Lee SH (2018). Tumor evolution and drug response in patient-derived organoid models of bladder cancer. Cell.

[13] Mullenders J (2019). Mouse and human urothelial cancer organoids: a tool for bladder cancer research. Proc. Natl. Acad. Sci. USA.

[14] Knuchel R, Hofstadter F, Jenkins WE, Masters JR (1989). Sensitivities of monolayers and spheroids of the human bladder cancer cell line MGH-U1 to the drugs used for intravesical chemotherapy. Cancer Res..

[15] Yoshida T (2015). High-dose chemotherapeutics of intravesical chemotherapy rapidly induce mitochondrial dysfunction in bladder cancer-derived spheroids. Cancer Sci..

[16] Lamy P (2016). Paired exome analysis reveals clonal evolution and potential therapeutic targets in urothelial carcinoma. Cancer Res..

[17] Ben-David U (2017). Patient-derived xenografts undergo mouse-specific tumor evolution. Nat. Genet..

[18] Clappier E (2011). Clonal selection in xenografted human T cell acute lymphoblastic leukemia recapitulates gain of malignancy at relapse. J. Exp. Med..

[19] Eirew P (2015). Dynamics of genomic clones in breast cancer patient xenografts at single-cell resolution. Nature.

[20] Liu Y (2019). The androgen receptor regulates a druggable translational regulon in advanced prostate cancer. Sci. Transl. Med..

[21] Lam H-M (2014). Targeting GPR30 with G-1: a new therapeutic target for castration-resistant prostate cancer. Endocr. Relat. Cancer..

[22] Kim D (2013). TopHat2: accurate alignment of transcriptomes in the presence of insertions, deletions and gene fusions. Genome Biol..

[23] Chou J (2013). Phenotypic and transcriptional fidelity of patient-derived colon cancer xenografts in immune-deficient mice. PLoS ONE.

[24] Anders S, Pyl PT, Huber W (2015). HTSeq–a Python framework to work with high-throughput sequencing data. Bioinformatics.

[25] Robinson MD, McCarthy DJ, Smyth GK (2010). edgeR: a Bioconductor package for differential expression analysis of digital gene expression data. Bioinformatics.

[26] Kamoun A (2020). A consensus molecular classification of muscle-invasive bladder Cancer. Eur. Urol..

[27] Thorvaldsdóttir H, Robinson JT, Mesirov JP (2013). Integrative Genomics Viewer (IGV): high-performance genomics data visualization and exploration. Br. Bioinform..

[28] Tate JG (2019). COSMIC: the Catalogue Of Somatic Mutations In Cancer. Nucl. Acids Res..

[29] Rogers MF (2018). FATHMM-XF: accurate prediction of pathogenic point mutations via extended features. Bioinformatics.

[30] Xylinas E (2016). An Epigenomic Approach to improving response to neoadjuvant cisplatin chemotherapy in bladder cancer. Biomolecules..

[31] Menendez-Gonzalez JB (2019). Gata2 as a crucial Regulator of stem cells in adult hematopoiesis and acute myeloid leukemia. Stem Cell Rep..

[32] Renault VM (2009). FoxO3 regulates neural stem cell homeostasis. Cell Stem Cell.

[33] Le Grand F, Jones AE, Seale V, Scimè A, Rudnicki MA (2009). Wnt7a activates the planar cell polarity pathway to drive the symmetric expansion of satellite stem cells. Cell Stem Cell.

[34] Pera MF (2004). Regulation of human embryonic stem cell differentiation by BMP-2 and its antagonist noggin. J. Cell Sci..

[35] Clémot M, Sênos Demarco R, Jones DL (2020). Lipid mediated regulation of adult stem cell behavior. Front. Cell Dev. Biol..

[36] Wang B (2018). Phospholipid remodeling and cholesterol availability regulate intestinal stemness and tumorigenesis. Cell Stem Cell.

[37] Duex JE (2018). Functional impact of chromatin remodeling gene mutations and predictive signature for therapeutic response in bladder cancer. Mol. Cancer Res..

[38] Attar N, Kurdistani SK (2017). Exploitation of EP300 and CREBBP lysine acetyltransferases by cancer. Cold Spring Harb. Perspect. Med..

[39] Takeuchi A (2012). p300 mediates cellular resistance to doxorubicin in bladder cancer. Mol. Med. Rep..

[40] van Kessel KEM, Zuiverloon TCM, Alberts AR, Boormans JL, Zwarthoff EC (2015). Targeted therapies in bladder cancer: an overview of in vivo research. Nat. Rev. Urol..

[41] Winters BR (2018). Mechanistic target of rapamycin (MTOR) protein expression in the tumor and its microenvironment correlates with more aggressive pathology at cystectomy. Urol. Oncol..

[42] Druker BJ (1996). Effects of a selective inhibitor of the Abl tyrosine kinase on the growth of Bcr-Abl positive cells. Nat. Med..

[43] Solit DB (2006). BRAF mutation predicts sensitivity to MEK inhibition. Nature.

[44] Durand RE (1981). Flow cytometry studies of intracellular adriamycin in multicell spheroids in vitro. Cancer Res..

[45] Mehta G, Hsiao AY, Ingram M, Luker GD, Takayama S (2012). Opportunities and challenges for use of tumor spheroids as models to test drug delivery and efficacy. J. Control Release..

[46] Marusyk A (2016). Spatial proximity to fibroblasts impacts molecular features and therapeutic sensitivity of breast cancer cells influencing clinical outcomes. Cancer Res..

[47] Ben-David U, Beroukhim R, Golub TR (2019). Genomic evolution of cancer models: perils and opportunities. Nat. Rev. Cancer..

