# Supplementary material for: A bladder cancer patient-derived xenograft displays aggressive growth dynamics in vivo and in organoid culture
Source: Sci Rep. 2021 Feb 25;11:4609. doi: 10.1038/s41598-021-83662-7 (PMC7907272; doi:10.1038/s41598-021-83662-7)
Supplement: Supplementary file 1 — Supplementary Information 1. [file 41598_2021_83662_MOESM1_ESM.pdf]

A bladder cancer patient-derived xenograft displays aggressive growth dynamics *in vivo* and in organoid culture

Elise Y. Cai[1], Jose Garcia[2], Yuzhen Liu[1], Funda Vakar-Lopez[3], Sonali Arora[1], Holly M. Nguyen[2], Bryce Lakely[2], Lisha Brown[2], Alicia Wong[1], Bruce Montgomery[4], John K. Lee[1,4], Eva Corey[2], Jonathan L. Wright[2,5*], Andrew C. Hsieh[1,4*], Hung-Ming Lam[2*]

1. Division of Human Biology, Fred Hutchinson Cancer Research Center, Seattle, WA, USA
2. Department of Urology, University of Washington School of Medicine, Seattle, WA, USA
3. Department of Pathology, University of Washington School of Medicine, Seattle, WA, USA
4. Department of Medicine, University of Washington School of Medicine, Seattle, WA, USA
5. Division of Public Health Sciences, Fred Hutchinson Cancer Research Center, Seattle, WA, USA

Figure S1

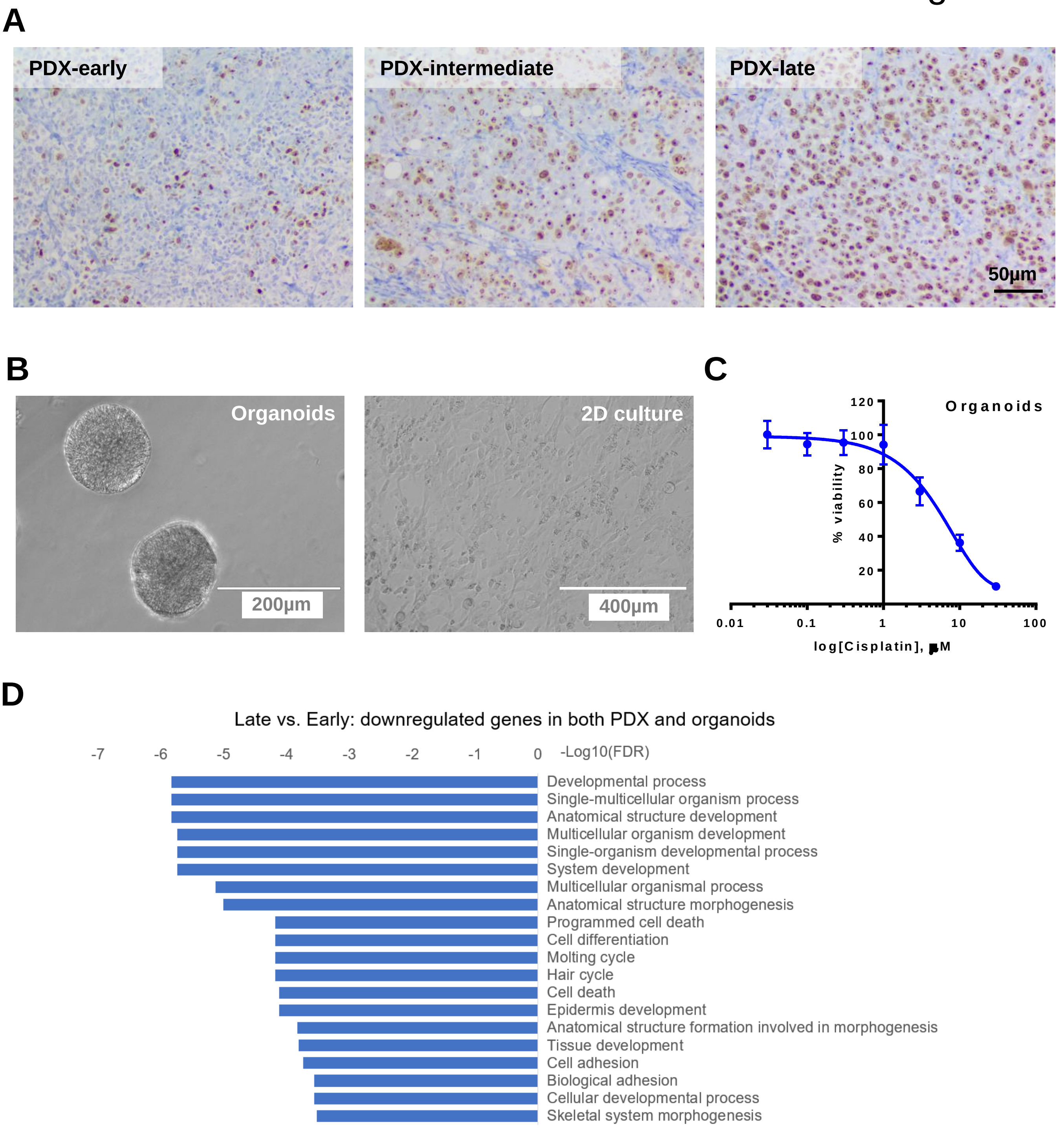

Figure S1. Characterization and gene pathway analysis in CoCaB 1 models. A) Representative bright field images of CoCaB 1 PDX and organoids, late passages. Scale bar represents 200 µm for PDX and 400 µm for 2D culture. **B)** Representative immunohistochemistry staining of Ki67 in early, intermediate, and late passage PDX demonstrates elevated Ki67 levels in later passages. Magnification 200x. Scale bar represents 50 µm. **C)** Cisplatin response on early passage organoids. **D)** Ingenuity Pathway Analysis of 306 genes commonly downregulated in late vs. early passages in both CoCaB 1 PDX and organoids.

A

	Consensus class	Separation level
Patient tumor	Ba/Sq	0.67
PDX-Early	Ba/Sq	0.92
PDX-Early	Ba/Sq	0.93
PDX-Early	Ba/Sq	0.64
PDX-Late	Ba/Sq	0.55
PDX-Late	Ba/Sq	0.63
PDX-Late	Ba/Sq	0.51
Organoid-Early	Ba/Sq	0.49
Organoid-Early	Ba/Sq	0.50
Organoid-Late	NE-like	0.59
Organoid-Late	NE-like	0.51

B

	Patient tumor	PDX-Early	PDX-Early	PDX-Early	PDX-Late	PDX-Late	PDX-Late	Organoid-Early	Organoid-Early	Organoid-Late	Organoid-Late
Basal markers											
KRT5	11.6	11.7	9.3	7.2	4.7	5.8	3.7	6.5	7.2	0.6	0.4
KRT14	10.7	11.9	9.9	11.0	4.9	6.8	4.0	8.7	10.0	0.6	0.4
KRT17	6.0	8.3	7.0	7.8	5.4	4.7	5.0	6.2	5.9	2.5	3.3
KRT6A	6.0	5.1	5.0	0.9	4.0	4.7	3.8	0.4	0.3	0.1	0.0
Luminal markers											
GATA3	7.1	7.7	7.0	7.1	7.1	7.1	7.0	7.4	7.3	5.7	5.3
FOXA1	0.0	1.7	3.3	0.2	0.3	0.8	1.2	0.5	0.6	0.3	0.9
NE markers											
SYP	3.0	2.9	2.9	2.7	2.8	2.5	2.7	2.8	2.6	3.2	2.4
CHGA	0.3	1.3	1.3	0.3	0.6	0.6	0.6	0.9	0.4	0.0	0.4
CHGB	2	3.7	3.1	3	2.4	2.4	2.4	3.4	3.3	1.9	1.9

Figure S2. Molecular subtype characterization of CoCaB 1 models. A) Molecular subtype analysis based on consensus subtype classification revealed subtype assignment in each CoCaB 1 models and passages. The separation level ranges from 0 to 1 and gives a measure of how a sample is representative of its consensus class. **B)** RNAseq analysis showing log2(TPM) values of the marker transcript levels in the patient tumor, PDX models, and organoids models. Ba/Sq, basal/squamous; NE-like, neuroendocrine-like.

Figure S3

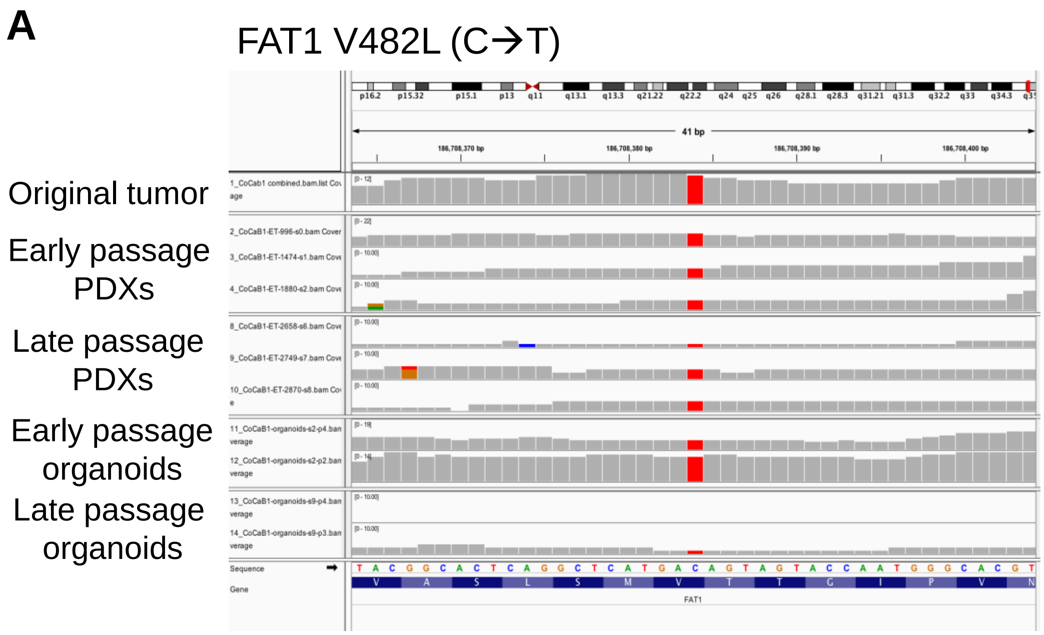

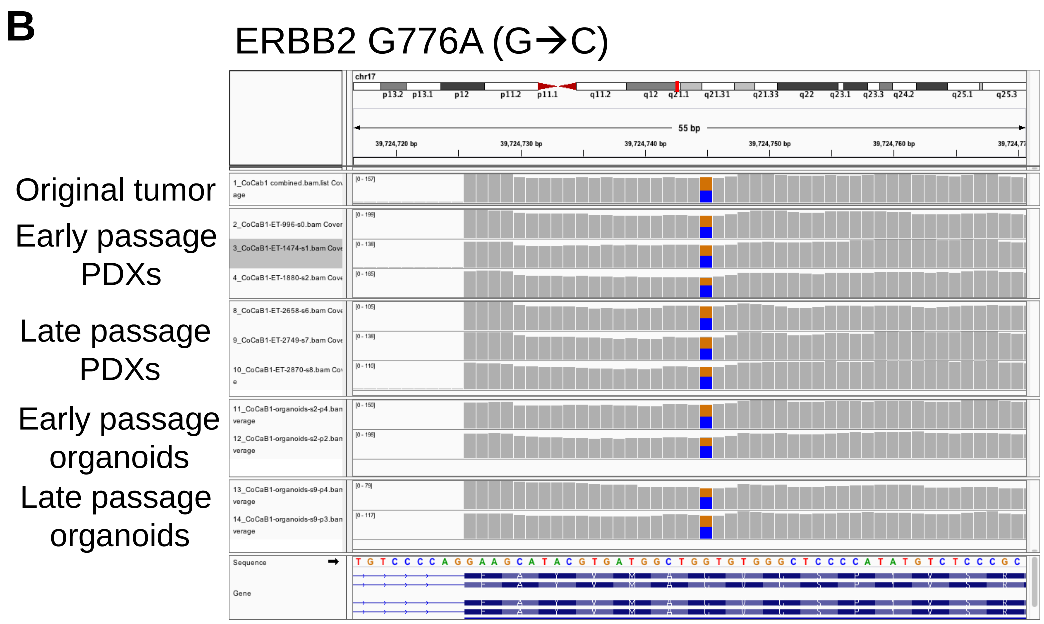

Figure S3. Missense mutations in patient tumor are maintained in PDX and organoids. A-B) IGV coverage tracks (27) of sequencing alignments demonstrate preservation of representative A) homozygous (FAT1 V482L) and B) heterozygous (ERBB2 G776A) missense mutations from original patient tumor through to late passage organoids.
